# The future of *Microbial Biotechnology*

**DOI:** 10.1111/1751-7915.13920

**Published:** 2021-10-06

**Authors:** Lawrence P. Wackett

**Affiliations:** ^1^ University of Minnesota St. Paul MN 55108 USA

Great strides have been made with regard to the one gene‐one enzyme‐one function paradigm in microorganisms. Indeed, biotechnology largely grew up on single gene cloning and overexpression – think insulin, erythropoietin or proteins rendering plants resistant to herbicides. This has been a lucrative enterprise. Recombinant human insulin, produced in microbial hosts, is medically superior to animal‐derived insulin, where sequence differences could cause undesirable immunoresponses.

Since those times, the synthesis and expression of multiple genes and functions has become readily attainable. The price of DNA synthesis is dropping considerably and new research advances in the gene synthesis industry will lower costs further (Eisenstein, 2021). This will allow the synthesis of artificial operons and other larger units of genomes. Gene multiplexing methods provide for laboratory evolution via combinatorial gene mixing. This has opened the door to ambitious genomic engineering projects.

To accomplish this broader vision of multifunctional engineering, there must be a corresponding revolution in better understanding gene interplay for phenotypes involving multiple genes. This is sometimes referred to as complex traits. By way of example, microbes naturally and constantly must respond to stresses of temperature, osmotic balance, desiccation and other environmental changes. The responses typically require multiple systems and consequently involve complex genetic interactions. Many components, such as various heat shock proteins, have been studied in isolation under one set of changing conditions. Increasingly, heat shock proteins are revealed to be involved in multiple stress responses.

Gene interplay is ripe for deeper understanding via machine learning approaches (Cai et al., 2021; Shah et al., 2021). This can take advantage of the burgeoning genomic and transcriptomic data, that is too complex for humans to analyse meaningfully by manual methods. This approach can highlight important genes in certain stress conditions, the interactions between genes, and how different microbes have evolved different strategies for dealing with changes.

A counterargument might be that methods like random forest tree building, or even mutating codes, give outputs that appear as a blackbox to humans. That is, we see a result, but cannot follow how the machine arrived at that endpoint. This might seem disconcerting at first. However, most scientists believe in the notion that the most important results emerge from good hypotheses. And good hypotheses regarding the most complex microbial processes are hard to come by. In that context, I foresee a beautiful machine‐human duality in which machines process large datasets, unexpected gene interactions emerge, and humans use that new information to devise new hypotheses and the key experiments to test them.

Some envision a further step into the machine world. Recently, biotech industries have expressed significant concerns about the irreproducibility of impactful biotechnology research results (Challenges in reproducible results). Most believe the problem largely emanates from small methodological inconsistencies that are not adequately communicated in the respective Materials and Methods sections of published articles. One proposed solution is the use of more automated methods run by standard computer code (Leman et al., 2021). Fortunately, for those of us dealing with microorganisms, bacterial cultivation and subsequent manipulations are more likely to prove reproducible than experiments conducted with colonies of lab rats. Still, automated microbial batch growth and robotic microtiter well screening are becoming much more common in microbiology laboratories. One can only expect that we will increasingly rely on machine methods for both enhancing reproducibility and increasing our research throughput. The machine‐human‐microbe axis is upon us, and with it, the future looks bright.
